# Can We Standardize Clinical Functional Neuroimaging Procedures?

**DOI:** 10.3389/fneur.2018.01153

**Published:** 2019-01-08

**Authors:** Roland Beisteiner, Cyril Pernet, Christoph Stippich

**Affiliations:** ^1^Department of Neurology, High Field MR Center, Medical University of Vienna, Vienna, Austria; ^2^Center for Clinical Brain Sciences, The University of Edinburgh, Edinburgh, United Kingdom; ^3^Department of Neuroradiology, University Hospital Zürich, Zurich, Switzerland

**Keywords:** functional MRI, clinical application, standardization, patients, neuroimaging

## Abstract

In recent years, the interest in clinical applications of functional neuroimaging techniques like functional Magnetic Resonance Imaging (fMRI) or modern Magneto- or Electro- Encephalography (MEG-EEG) has steadily grown as have discussions about possible standardizations of these methodologies. The modern techniques allow non-invasive localization of essential brain functions with the potential to extend or even replace invasive clinical technologies (1–4). The focus of this article is to discuss standardization options in using functional MRI for clinical cases, mostly in the context of medical decision aid for planning treatment (radiotherapy and surgery).

## What are the Problems for Standardizing Clinical Functional Neuroimaging Procedures?

Functional techniques are complicated and require technical, methodological, and neurophysiological expert knowledge. For patient investigations, specific patient expertise, and clinical knowledge are also mandatory. Quite evidently, methodological assumptions as developed for healthy subjects and implemented in standard software packages may not always be valid for distorted and pathological brains. There are specific problems for the various clinical populations and also for defining the functional status of an “individual brain” (as opposed to a “group brain” in group studies). Whilst fMRI has been used successfully in research for healthy subjects and patient groups (group brains), the situation is complicated for using fMRI as a clinical tool for individual patients. Here, patient and disease variability add to the complexity of fMRI. When imaging patients, every brain is different, not just among patients but also for the same patient over time (5, 6) as pathological brains can change rapidly (7) and their function depends on disease stage. In addition, every pathology (even within the same disease) is different in type, location, extent, and pathophysiological consequences (e.g., concerning effects on the hemodynamic response function HRF)—this has to be considered for functional imaging protocols. Due to this complexity, current clinical functional imaging protocols vary considerably across institutions in non-standardized ways. However, despite this situation, recent reviews of fMRI for presurgical evaluation of patients with epilepsy or brain tumors recommend the application of fMRI in certain contexts showing probable or possible usefulness for patients [levels B and C, (8), (9)]. In this situation and since procedural variability immediately affects validity and repeatability of fMRI results, a burning question for clinical functional neuroimaging is: can we standardize clinical fMRI procedures and to what extent?

At this point it is important to realize that even when focusing on healthy subject settings, it is difficult to unambiguously define best practice procedures. Consequently, the recent COBIDAS report [Best Practices in Data Analysis and Sharing in Neuroimaging using MRI, (10)] concentrates on recommendations for data reporting and little on best practice for experimental design, data acquisition, and preprocessing issues. Only for statistical modeling more detailed best practice recommendations are given. Considering the large body of existing clinical research, we believe that within a clinical context, some experimental and acquisition recommendations—which might be helpful over most patient populations and investigational systems—can also be given. With task-based fMRI, the most important clinical functional neuroimaging application to date, the major problems may be categorized in 2 classes: “Patient related challenges” and “Methodological challenges.” To approach a standardization of clinical fMRI, the first step is to identify the most important issues within every class, which are accessible for defining a procedural standard. In the following we suggest such starting points for procedural standardizations, depending on the problem class.

## Patient Related Challenges

Patient challenges in clinical fMRI particularly concern the clinical patient state (e.g., paresis, neglect, aphasia) which may largely differ despite having identical diagnoses. Patient state may even differ within the same patient from day to day. Besides patient state, issues with scanning patients concern performance level (intra-investigational compliance may change rapidly), ability to support restriction of head movements (in patients head movement artifacts may be very large), the effect of brain pathology on the MRI signal (variability of contrast to noise ratio, limitations for inter-image registration, and analysis) as well as running medication (e.g., antidepressants, tranquilizers). All those factors can interfere with and lead to widely different results and interpretation of functional signals. This may affect important diagnostic questions like “where are the essential activations located precisely in the given, distorted pathological brain?” To approach a standardization for patient related problems, the authors suggest the following issues for defining a standard:

(A) Requirements for the patient:

(1) Define a minimum level of vigilance and compliance. (2) Define a minimum performance level (e.g., for language task x, minimum required language capabilities are….).

(B) Requirements for patient handling:

(1) Define a minimum standard for patient preparation (e.g., adaptation to the system, training of tasks). (2) Define a minimum performance control (e.g., monitoring of language or motor output). (3) Define adequate procedures for clinical head fixation (high restriction efficiency offers the best imaging option but freeing oneself in case of emergency has to be considered).

## Methodological Challenges

For clinical fMRI standardizations, problems concerning infrastructure and methodology are also highly important. Here, profound knowledge and local validation of the hard- and software used is essential. Many of the methodological problems are reasons why the techniques have not yet found a broad way into routine clinical work (11). The following issues might be accessible for defining a standard:

(A) Requirements for data recording:

(1) What is the minimum equipment necessary (e.g., concerning MR systems, medically approved stimulus delivery systems, patient monitoring, and response systems)? (2) What are the minimum requirements for clinically feasible and informative functional neuroimaging protocols? This includes standards for protocol development, testing, optimization, task definitions, and design definitions (e.g., use only published protocols, check advantages of blocked vs. event related designs, check advantages of resting state vs. task-based fMRI, check advantages of adding arterial spin labeling (ASL) to gain additional functional information from a different perspective). (3) Which MRI sequence characteristics should be recommended to allow comparability over many MR systems (e.g., spatial resolutions, TR values, use of multiband factors, number of volumes, and minimum scanning time etc.)? This may result in concrete suggestions of sequence models which might be immediately copied for resting state fMRI, task based fMRI, anatomy, DTI etc.

(B) Requirements for data analysis:

(1) What are the acceptable post processing steps and how should a “clinically significant” brain activation be defined? As indicated above, model assumptions developed for healthy brains may not work and a false positive risk has to be balanced against clinically critical false negatives (12). In general, data analyses with minimized model assumptions seem most promising. Recommendations might also include the latest options to remove residual head motion effects and the simultaneous and coparative use of various data analysis approaches (e.g., the classical model-based approaches and those with minimized assumptions to help differentiation of artifacts from low level activations). (2) How should the formats for data presentation to the clinicians be—considering that “final clinical images” always include an interpretation?

## How is the Current Situation of Standardization Efforts?

For experts in clinical fMRI, the topic is not a new one and has been discussed for decades within dedicated societies (www.asfnr.org, www.oegfmrt.org, www.humanbrainmapping.org). However, based on recent critics about replicability/reproducibility of neuroimaging data (13–15), this discussion spread and has meanwhile become a major topic of larger imaging societies within the fields of brain mapping, clinical neurosciences, and radiology. New efforts to standardize functional neuroimaging protocols have been launched particularly by the Organization for Human Brain Mapping (OHBM). For MRI based neuroimaging research the COBIDAS report has been published (10) and a recommendation on MEG/EEG is in preparation (16). The COBIDAS report introduces recommendations within seven areas of MRI neuroimaging: (1) experimental design reporting, (2) image acquisition reporting, (3) preprocessing reporting, (4) statistical modeling, (5) results reporting, (6) data sharing, and (7) reproducibility. From these areas the general recommendations for procedure reporting may well be applicable for clinical research and diagnostic patient investigations—although specific extensions for clinical populations will be required as outlined above. Similar approaches focused on clinical demands currently exist on local levels (e.g., Neuroimage WING project Austria, www.i-med.ac.at/mypoint/thema/678651.html), on specific topics [European Network for Brain Imaging of Tumors (ENBIT, www.enbit.ac.uk)] or on specific diseases (e.g., Magnetic Resonance Imaging in Multiple Sclerosis, www.magnims.eu). Larger international standardization initiatives are currently under discussion with an intention to also increase overlap between radiological societies and functional imaging societies. These initiatives are mostly focused on neuroimaging with healthy subjects, however they clearly indicate the need for extension of these discussions to the field of clinically applied neuroimaging.

## Conclusion

In conclusion, we think that current initiatives are helpful to spread attention on repeatability, reproducibility, and replicability issues from smaller circles to larger communities and societies. The goal is to define standards for neuroimaging wherever possible. We argue for increased awareness and critical consideration of the specific problems with patients and clinical environments in the context of clinical fMRI. Given the broad range of disease related and methodological variability, defining procedural clinical standards is an utmost difficult enterprise. Helpful recommendations may need to be relative (e.g., depending on disease and patient state) and not absolute. However, for the most important clinical functional neuroimaging method—task based clinical fMRI—developing recommendations for the patient- and methodological challenges described above seems feasible and is highly desirable to move the technology into the clinical realm.

## Author Contributions

RB designed the manuscript concept, all authors participated in manuscript writing and editing.

### Conflict of Interest Statement

The authors declare that the research was conducted in the absence of any commercial or financial relationships that could be construed as a potential conflict of interest.
